# Recurrent lung infection after slipped gastric band in a patient with morbid obesity

**DOI:** 10.1093/jscr/rjag721

**Published:** 2026-08-20

**Authors:** Akshita Patel, Justine Izah, Daniel Henley, Alexander P Lynch, Francis J Podbielski

**Affiliations:** Department of Surgery, Ascension Saint Joseph Hospital, 2900 North Lake Shore Drive, Chicago, IL 60657, United States; Department of Surgery, Ascension Saint Joseph Hospital, 2900 North Lake Shore Drive, Chicago, IL 60657, United States; Department of Surgery, Ascension Saint Joseph Hospital, 2900 North Lake Shore Drive, Chicago, IL 60657, United States; Department of Biological Sciences, Creighton University, 2500 California Plaza, Omaha, NE 68178, United States; Department of Surgery, Ascension Saint Joseph Hospital, 2900 North Lake Shore Drive, Chicago, IL 60657, United States

**Keywords:** obesity, slipped gastric band, recurrent lung abscess, aspiration

## Abstract

We report the case of a 43-year-old male with a history of gastroesophageal reflux, obstructive sleep apnea, and morbid obesity who underwent gastric band placement in 2007, with left thoracotomy in 2012 for a loculated empyema. He now presents in acute respiratory distress with fetid-smelling sputum, a leukocytosis of 19.5 K, and a complex left lower lobe lung abscess on imaging. He underwent a redo left thoracotomy, partial rib resection, and drainage/debridement of the lung abscess. Postoperatively, he experienced ongoing poor oral intake due to high-grade stenosis caused by a slipped gastric band, noted on an upper gastrointestinal series. He underwent removal of the gastric band with robotic assistance by the bariatric service and was subsequently discharged after his respiratory status and oral intake improved. Here, we highlight that recurrent lung infections in bariatric surgery patients secondary to chronic reflux and poor dietary compliance mandate long-term surveillance and thorough investigation.

## Background

Obesity is known to be multi-factorial, including but not limited to metabolic syndrome, excessive caloric intake, and lack of physical activity [1, 2]. Various treatment modalities, both medical and surgical, are available for the management of this problem. Bariatric surgery strategies are divided into two categories: restrictive and malabsorptive. Restrictive bariatric surgeries include gastric bands and sleeve gastrectomy. Malabsorptive bariatric surgeries include Roux-en-Y gastric bypass and biliopancreatic diversion with duodenal switch. Gastric band placement, a form of restrictive surgery, has fallen out of favor due to the high complication profile reported in the literature, including band slippage, band erosion, and port malfunction [3]. Less commonly, gastric band placement can be associated with pulmonary complications such as lung abscess, empyema, asthma exacerbation, interstitial lung disease, and bronchiectasis [4, 5]. We discuss a unique case of recurrent pneumonia, leading to lung abscess, secondary to aspiration, in a morbidly obese patient with a slipped gastric band over 10 years after placement.

## Case

**Figure 1:**
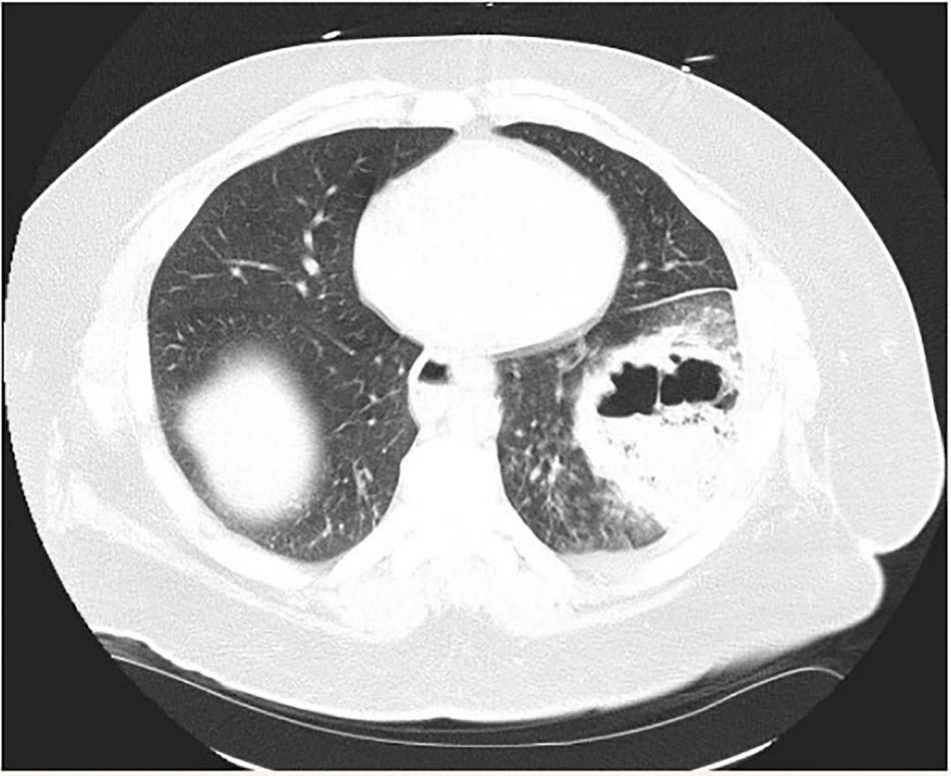
A CT scan showing a large lung abscess in the superior segment of the lower lobe of the left lung.

**Figure 2:**
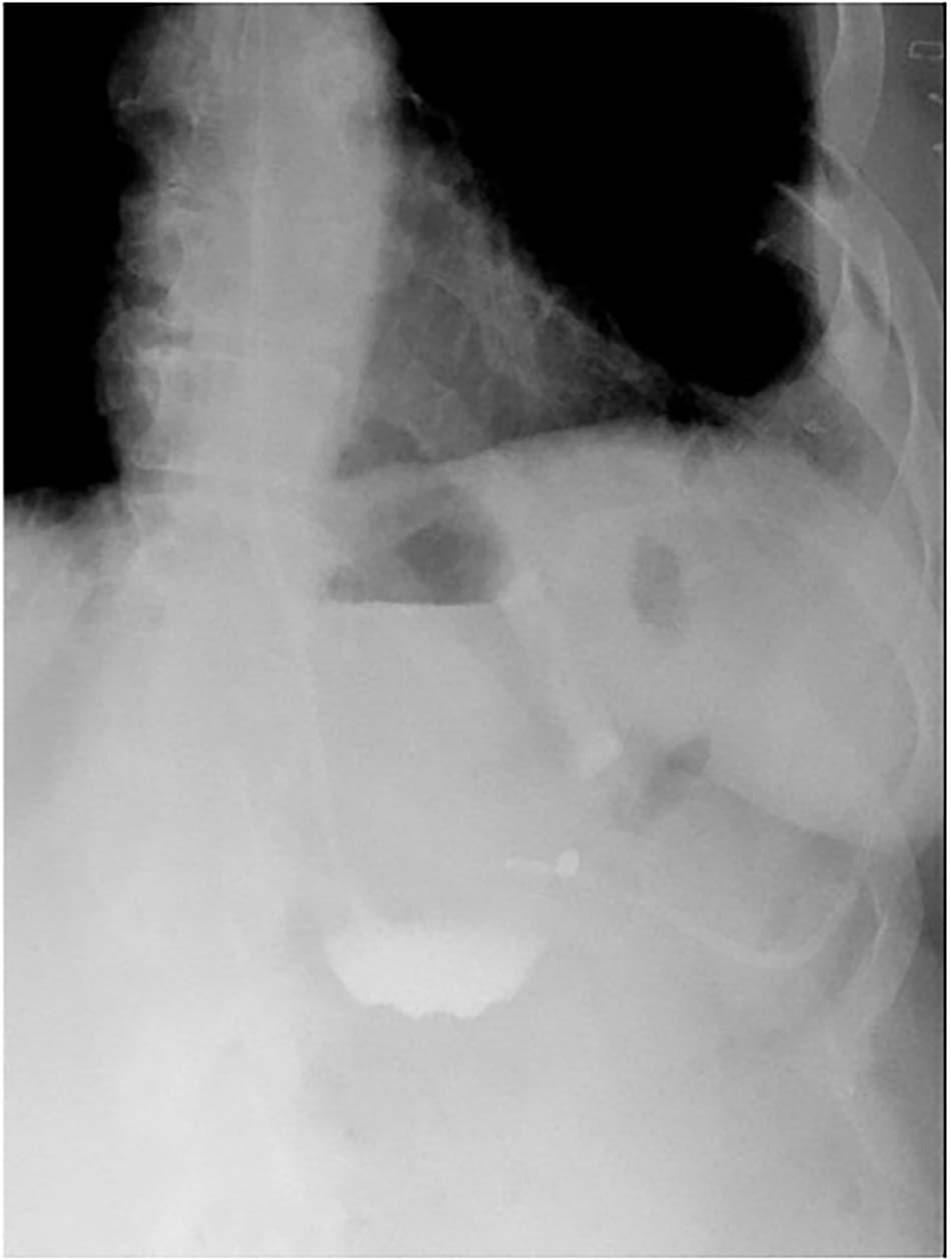
An upper gastrointestinal series showing a slipped gastric band causing high-grade stenosis.

**Figure 3:**
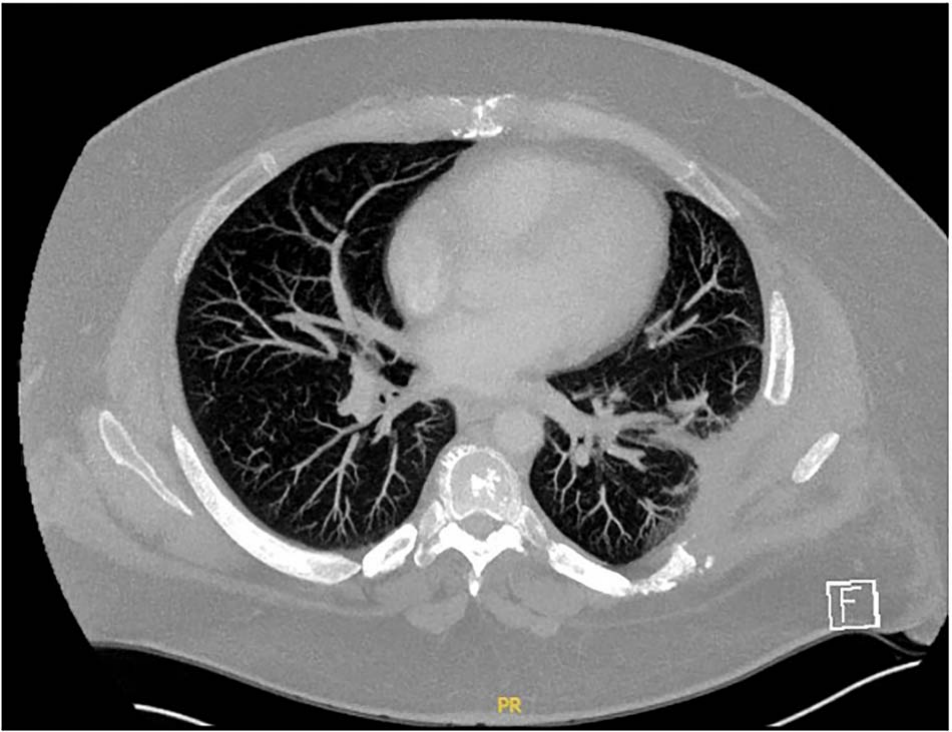
CT scan from May 2023, 5 months after treatment, showing a defect in the chest wall with soft tissue filling and excellent healing of the lung parenchyma.

Patient is a 43-year-old male with a past medical history of obstructive sleep apnea, chronic gastroesophageal reflux disease, and morbid obesity, for which he underwent laparoscopic gastric band placement in 2007. He was initially lost to follow-up, but presented in 2012 with left thoracic empyema, thought to be secondary to recurrent aspiration; a left thoracotomy and lung decortication were performed at that time. He again presented in 2022 with acute respiratory distress with a 4-day productive cough and fetid-smelling sputum. On admission, he was febrile, tachycardic, and required supplemental oxygen support. Laboratory workup was significant for leukocytosis of 19.5 K. A computed tomography (CT) scan of the chest showed a large lung abscess essentially replacing the superior segment of the left lower lobe of the lung (Fig. 1). He underwent a redo left thoracotomy with partial sixth rib resection and lung abscess debridement. Dense adhesions were expected intraoperatively due to prior infection and surgery; therefore, using the CT scan as a guide, the abscess was located beneath the scapula and inferior to the sixth rib, and an incision was planned on the sixth rib for adequate access and visualization. Once identified, the abscess cavity was debrided, drains were placed, and the tissues were loosely approximated to provide drainage of the significant subcutaneous tissue, given his obesity. The patient’s postoperative course was complicated by ongoing poor oral tolerance, for which he underwent an upper gastrointestinal series on post-operative day (POD) #8 (Fig. 2). This revealed a slipped gastric band causing high-grade stenosis of the proximal stomach. He then underwent a nasogastric tube placement under anesthesia for enteral feedings with subsequent removal of the gastric band and lysis of adhesions with robotic assistance on POD #11. His nausea and oral intake significantly improved postoperatively, and he was subsequently discharged on POD #22 on a regular diet. A repeat CT chest scan from May 2023, 5 months after the redo thoracotomy, revealed soft tissue filling the defect created by the resection of the sixth rib with excellent healing of the lung parenchyma (Fig. 3).

## Discussion

Bariatric surgery has become an integral part of the management of morbid obesity. Gastric band placement was previously a commonly performed bariatric procedure; however, several complications have been reported, making it a less attractive option. Frequent complications reported in the literature include pouch enlargement, band slippage, band erosion, port site infection, port malfunction, and inadequate weight loss [3]. Less commonly, pulmonary complications such as lung abscess and empyema have also been reported in the literature. Several reports have identified patients who developed pulmonary complications in the time frame of 1 month to 3 years after the index operation. Additionally, retrospective studies have also reported a strong association between gastric band placement and pulmonary complications [4, 6]. For instance, a retrospective study by Avriel *et al*. evaluated 2100 patients who had undergone gastric band placement. Of those, 30 patients developed respiratory complications, with aspiration pneumonia (19 patients) and lung abscess (four patients) being the most common. These studies identified gastric band removal as the main mode of treatment, and those who refused the operation continued to experience pulmonary complications. Furthermore, studies have reported an increased rate of respiratory distress with the gastric band when compared to different bariatric procedures. Shitrit *et al*. reported an overall higher complication rate with gastric band compared to sleeve gastrectomy (7.13% and 4.4%, respectively), a significantly higher rate of respiratory complaints of morning and postprandial cough compared to sleeve gastrectomy (59.6% vs 12.3% and 58% vs 10.5%, respectively) [7].

We report a unique case of a morbidly obese patient who developed recurrent lung abscess from a slipped gastric band, secondary to aspiration, over 10 years after it was placed. This is the first case reported with said complication in this time frame. The case clearly illustrates that long-term pulmonary complications from recurrent aspirations pose a high risk of morbidity and mortality in patients who have undergone gastric band placement. This risk is increased particularly in patients with poor follow-up compliance. Therefore, proper evaluation with contrast GI studies and pulmonary imaging is mandated, along with prompt loosening/removal of the gastric band, at the initial presentation, to minimize the risk of ongoing aspiration. Furthermore, long-term surveillance is mandated to optimize outcomes and minimize complications.
